# Body Mass Index and the Literacy on Obesity in Relation to Media Following

**Published:** 2018-08

**Authors:** Goran BELOJEVIC, Lilijana SOKOLOVA DJOKIC, Biljana GLIGOROVA, Ines BANJARI, Marko STOJANOVIC, Dusica STOJANOVIC

**Affiliations:** 1. Institute of Hygiene and Medical Ecology, Faculty of Medicine, University of Belgrade, Belgrade, Serbia; 2. College of Professional Studies in Education of Teachers and Trainers, Subotica, Serbia; 3. Faculty of Pedagogy, St Kliment Ohridski, Bitola, Republic of Macedonia; 4. Dept. of Food and Nutrition Research, Faculty of Food Technology, Josip Juraj Strossmayer University of Osijek, Osijek, Croatia; 5. Institute of Public Health, Nis, Serbia; 6. Dept. of Hygiene and Human Ecology, Institute of Public Health, Faculty of Medicine, University of Nis, Nis, Serbia

**Keywords:** Body mass index, Obesity, Literacy, Internet

## Abstract

**Background:**

The aim of this study was to investigate the effects of TV, magazines, radio, and internet following on body mass index (BMI) and obesity-related literacy among adults.

**Methods:**

In this cross-sectional study, the subjects were recruited from an outpatient center in the city of Sombor, Serbia during Mar–Apr 2013. We collected data by a questionnaire from 657 (397 women; 59%) subjects, aged from 18 to 87 yr (Mean = 45; SD =14). The questionnaire consisted of personal data, body height and weight, frequency of television, radio, magazines and internet following and personal opinion on the impact of smoking, alcohol consumption, stress and physical inactivity on obesity.

**Results:**

Spearman’s rank correlation analysis showed that BMI increased with longer TV viewing with a very weak strength of the correlation (r=0.104; *P*=0.009) and decreased with more internet following with a weak strength of the correlation (r=−0.200: *P*<0.001). Multiple linear regression analysis revealed that only internet use had a significant independent effect on BMI. The frequency rise of internet following from “rare” to “often” and “every day” decreased BMI by 0.5 per each grade. Internet followers showed a significantly better knowledge of the importance of smoking (*P* = 0.003), alcohol consumption (*P*<0.001) and physical inactivity (*P*=0.004) for obesity in comparison to non-followers.

**Conclusion:**

Internet is the only media that independently and positively influence weight control and the literacy on obesity among adults.

## Introduction

Physical inactivity observed as the total “sit-time”, one of the major risk factors for obesity is commonly related to media following. For children and adolescents the internet use may be problematic (1) as well as the prolonged television (TV) viewing (2). For adults, TV viewing has been studied most extensively and shows linear correlation with the risk of becoming overweight/obese (3). This relationship stands also for obesity-related diseases. For example, for every two hours spent in watching TV, the risk of developing diabetes, heart disease, and early death increases by 20%, 15%, and 13%, respectively (4). Of a special scientific interest are social networking sites which are commonly daily used and associated with increased odds of skipping breakfast and consuming sugar-sweetened beverages and energy drinks (5). Another negative effect of media following on obesity prevention may derive from the advertisements directed towards energy-dense, nutrient-poor food (6, 7). The absence of advertising for unhealthy food on TV would bring normal weight to one in seven up to one in three obese children in the US (8). However, the teams of scientists from food and beverage industry continuously work on normalizing the brands of low nutritional value food and beverage, especially with adolescents’ culture. Internet and particularly online social networks are targets for these campaigns.

On the other side, media, including TV, magazines, radio, and internet are also easily reachable sources of competent information on obesity risk factors and the ways to prevent it. Thus, the effects of media following on obesity may be double-sided and balanced by the ratio between sedentary lifestyle and misleading advertisements and preventive activities based on proper information. Further, it is of scientific interest to compare the effects of following different media on weight control. Knowledge on obesity prevention may be investigated through attitudes on obesity, while healthy behavior is reflected through weight control, namely by body mass index (BMI).

Weight control and relevant attitudes of adults on obesity depend on media following (9), but there is sparse information on the comparative importance of specific media in this relationship. The aim of this study was to investigate the effects of TV, magazines, radio, and internet following on BMI and obesity-related literacy among adults. Additionally, we test if any media following independently affects BMI and how it is related to the health literacy on obesity.

## Methods

### Sample

The subjects were recruited from an outpatient center in the city of Sombor, Serbia, during Mar–Apr 2013. Out of 864 interviewed patients, 657 (397 women; 59%) or 76% agreed to participate in the study.

Informed consent was taken from the participants before and the study was approved by the university.

A study-specific questionnaire was self-completed by each participant in the outpatient center while waiting to be examined. Age range was 18 to 87 yr (45±14 yr). The participants were not informed about the aim of the study to avoid a recall bias. We reduced the number of questions in a closed questionnaire with offered answers on a simple four graded ordinal scale, based on our experience that for interviewing in such conditions the length of a questionnaire is decisive for a high response rate. It took just about five minutes to complete the questionnaire in order to avoid time pressure and the participants’ lack of concentration. The questionnaires were available during the whole work time of the outpatient clinic to enable full randomization.

### Questionnaire

The questionnaire consisted of personal data, body height and weight, frequency of media (TV, magazines, radio, and internet) following (0=never; 1=rarely; 2=often; 3=every day), and personal opinion on the impact of well-known obesity risk factors, i.e. smoking, alcohol consumption, stress and physical inactivity on obesity (no=0, little=1; moderate=2; great=3).

In our opinion the choice of offered answers on ordinal scales is appropriate, enabling the participants to easily decide which answer corresponds most closely to personal experience and knowledge. We consider such a questionnaire design valid and reliable in testing the established hypotheses.

### Statistical analysis

Beside descriptive statistics, a Spearman’s intercorrelation and a multiple linear regression analysis were performed with BMI as dependent variable and independent variables that appeared to be significant in a bivariate correlation analysis. In the non-parametric statistics, a Chi-square test was performed to compare the distribution of answers to the questions on obesity-related attitudes in relation to media following. Statistical analysis was performed using STATA statistical software, StataCorp, Tulsa,), with the chosen level of significance *P*=0.05.

## Results

Spearman’s inter-correlation matrix shows that BMI is significantly lower among women than men and increases with age with a weak strength of the correlation (r =0.293; *P*<0.001). Concerning media following, BMI increases with longer TV viewing with a very weak strength of the correlation (r=0.104; *P*=0.009) and decreases with more internet following with a weak strength of the correlation (r= −0.200: *P*<0.001). There are significant positive inter-correlations between TV, radio, magazines and internet following, with a very weak to weak strength of the correlations (r from 0.113 to 0.235; *P*<0.001). The comparatively strongest and negative correlation exists between the subjects’ age and internet following with a moderate strength of the correlation (r= −0.490; *P*<0.001) (Table 1).

**Table 1:** Intercorrelation matrix of body mass index (BMI) and relevant variables among adult outpatients of a Health Center in Sombor, Serbia (N=657; Spearman’s correlation; Correlation Coefficient/P)

***Variable***	***BMI***	***Sex***	***Age***	***TV***	***Radio***	***Magazines***	***Internet***
BMI	1.000	−0.206	0.293	0.104	0.038	0.048	−0.200
		< 0.001	< 0.001	0.009	0.402	0.275	< 0.001
Sex (1=male;2=female)		1.000	−0.065	−0.007	−0.095	−0.050	0.061
			0.099	0.870	0.037	0.257	0.170
Age			1.000	0.135	0.037	0.044	−0.490
				0.001	0.425	0.317	< 0.001
TV				1.000	0.235	0.235	0.113
					< 0.001	< 0.001	0.011
Radio					1.000	0.424	0.239
						< 0.001	< 0.001
Magazines						1.000	0.287
							< 0.001
Internet							1.000

Multiple linear regression analysis with BMI as a dependent variable and significant variables from inter-correlation matrix shows that with the increased frequency of internet following from “rare” to “often” and “every day” BMI decreases by 0.5 per each grade. Relationship between TV viewing and BMI is insignificant when controlled for sex, age, and internet following (Table 2).

**Table 2:** Multiple linear regression analysis with BMI as a dependent variable and significant variables from intercorrelation analysis among adult outpatients from the Sombor Health Center, Serbia (N =657)

***Variable***	***Unstandardized Coefficients***		***Standardized Coefficients***	***t***	***P***
	***B***	***Std. Error***	***Beta***		
(Constant)	25.843	1.279		20.202	< 0.001
	−1.889	0.397	−0.202	−4.761	< 0.001
Sex (1=male;2=female)					
Age	0.064	0.016	0.201	3.902	< 0.001
TV	0.475	0.305	0.068	1.561	0.119
Internet	0.461	0.199	−0.118	−2.311	0.021

Distribution of answers on the questions related to different risk factors for obesity in relation to internet following shows that followers have better knowledge on the importance of smoking (Chi-square=14.2; *P*=0.003), alcohol consumption (Chi-square= 23.1; *P*<0.001) and physical inactivity (Chi-square=13.2; *P*=0.004) for obesity in comparison to non-followers (Table 3).

**Table 3:** Distribution of answers on the questions of impact of smoking, alcohol consumption and physical inactivity on obesity in relation to internet following (N/%/)

***Internet Following***	***Impact of smoking***					***Chi-Square***	***P***
	***No***	***Little***	***Moderate***	***Great***	***Total***		
No	24 (25)	18 (19)	25 (26)	29 (30)	96 (100)		
Yes	38 (10)	69 (19)	117 (26)	139 (38)	363 (100)		
Total	62 (14)	87 (19)	142 (31)	168 (37)	459 (100)	14.2	0.003
Impact of alcohol consumption							
No	22 (23)	11 (12)	31 (33)	31 (33)	95 (100)		
Yes	25 (7)	60 (16)	123 (34)	159 (43)	387 (100)		
Total	47 (10)	71 (15)	154 (33)	190 (41)	482 (100)	23.1	< 0.001
Impact of physical inactivity							
No	1 (1)	4 (4)	29 (29)	65 (66)	99 (100)		
Yes	3 (1)	11 (3)	56 (14)	319 (82)	389 (100)		
Total	4 (1)	15 (3)	85 (17)	384 (79)	488 (100)	13.2	0.004

Their knowledge of the importance of stress for obesity is similar (Chi square=6.9; *P*=0.08; data not shown).

## Discussion

We showed that among the four basic media only internet following has a significant and positive effect on controlling weight gain and on obtaining better health literacy on risk factors for obesity, compared to non-followers. The Patient Protection and Affordable Care Act (10) defines health literacy as the degree to which an individual has the capacity to obtain, communicate, process, and understand basic health information and services to make appropriate health decisions.

Comparative advantage of internet over other media in individual improvement of health literacy includes the possibility to: explore several sources of information simultaneously, assess the quality of information, interact with the source of information, exchange experiences through online social networks, organize wide health literacy campaigns and deliver eHealth interventions to improve specific health literacy skills (11). Health information technology is a moving force of any health system where the patient becomes the main driver in his, personal health management. The personal health management is observed as a priority and as one of the key components in implementation of a public-oriented intervention for non-communicable diseases like obesity, as well as for enhancement of all health care systems, from the United States of America (12) to Europe (13). Additionally, WHO promotes effective use of the media in health promotion (14), i.e. the media can empower people for better health choices. However, privacy and security appear to be the main obstacles in faster adoption of health information technology (15).

Similar to our results of lower health literacy concerning obesity and higher BMI among internet non-followers compared to followers; adequate health literacy among women was more likely to rely on information obtained from the internet and to be related to a lower BMI than among those with a low health literacy (16). As shown in our study, older age is related both to a lack of internet access and increased BMI. Older age was more generally associated with higher rates of limited health literacy (17).

There are several limitations of our study. We relied on the subjects’ subjective assessment of their height and weight due to the inconvenience of measuring each participant in the waiting room of the outpatient center. According to the CAESAR project, an international co-operation to obtain anthropometric data from the populations of Europe and North America 11.2% of females and 12.0% of males underestimate their state of nourishment (18). Further, the questionnaire did not include more detailed questions related to the internet use or how often social network sites were visited. While the response rate of 76% was quite satisfactory there is a possibility of a recall bias or a confounding bias due to diseases that we did not control among the interviewed patients of the outpatient clinic.

## Conclusion

Contrary to children and adolescents for whom internet use is considered as problematic, for adults internet may have a positive effect on weight control and obesity-related literacy. The internet appears to be a very potent driver towards improved health literacy and empowered personal healthcare management. Globesity, as the nowadays escalating obesity epidemic is called, asks for more potent and urgent actions. Life-long learning, i.e. educating adults, especially elderly on the internet use should be considered as a potential additional approach in tackling the globesity.

## Ethical considerations

Ethical issues (Including plagiarism, informed consent, misconduct, data fabrication and/or falsification, double publication and/or submission, redundancy, etc.) have been completely observed by the authors.
